# Giant mucinous adenocarcinoma of the appendix: a case report

**DOI:** 10.1186/s13256-017-1385-1

**Published:** 2017-07-31

**Authors:** Sheng-Chun Dang, Ming-Ming Sun, Lu-Lu Liu, Abdul Malik, Jian-Xin Zhang, Yi-Yi Fan, Song Zou, Lei Cui, Jian-Guo Qu, Ji-Xiang Chen

**Affiliations:** Department of General Surgery, The Affiliated Hospital of Jiangsu University, No. 438 Jiefang Road, Zhenjiang, 212001 Jiangsu Province China

**Keywords:** Appendiceal mucinous adenocarcinoma, Appendiceal neoplasm, Appendectomy

## Abstract

**Background:**

Appendiceal mucinous adenocarcinoma is an extremely rare disease in clinical practice. Here, we report a case of unprecedented size that occupied the entire abdomen of a man.

**Case presentation:**

A 49-year-old Chinese Han man presented with symptoms of abdominal distension. During a computed tomography imaging examination, a cystic-solid mass that occupied his entire abdominal cavity was detected. During exploratory laparotomy, an appendiceal tumor in his abdominal-pelvic cavity measuring 27.6 × 14.2 cm was found, and he underwent tumor resection. The pathology of the tumor identified a well-differentiated appendiceal mucinous adenocarcinoma with mucin infiltrating into the soft tissue of the lump edge and omentum tissue. After surgery, our patient accepted intraperitoneal infusion chemotherapy. At present, he has had no recurrence for 15 months.

**Conclusions:**

To the best of our knowledge, the present case is the largest appendiceal mucinous adenocarcinoma reported. Surgical tumor resection is the preferred treatment for appendiceal mucinous adenocarcinoma. This is supplemented by chemotherapy which can further prolong survival.

## Background

Primary neoplasms of the appendix are very rare and histologically diverse. Tumors are broadly classified as colonic-type or mucinous adenocarcinoma, goblet cell adenocarcinoma, or neuroendocrine carcinoma [1]. Adenocarcinoma is considered to be the most common type of primary appendix cancer, accounting for 60% of all cases [2]. For mucinous adenocarcinoma of the appendix (MAA), the mean age at presentation is 60 years and no gender bias has been confirmed thus far [3]. Patients with MAA rarely present with specific clinical manifestations; therefore, appendiceal adenocarcinomas are usually found late or misdiagnosed. The majority of cases are diagnosed during intraoperative exploration.

## Case presentation

**Fig. 1:**
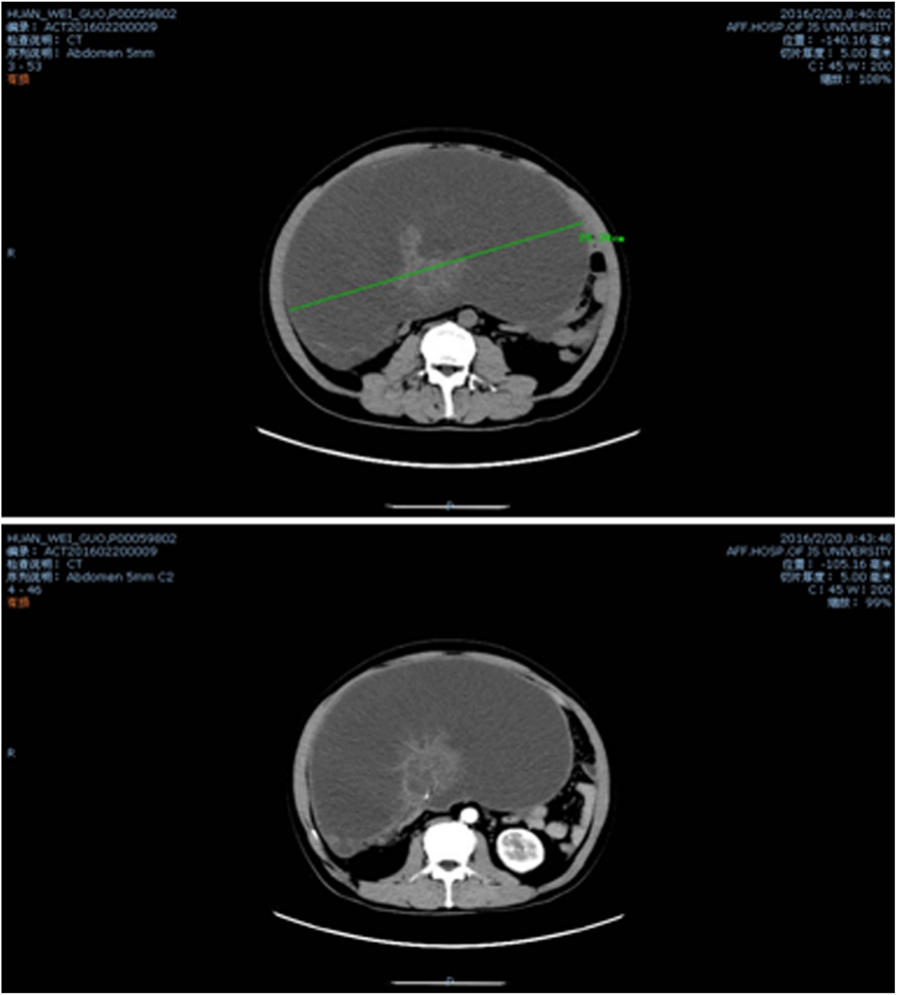
An abdomen computed tomography scan revealed a giant cystic mass in the abdominal-pelvic cavity

**Fig. 2:**
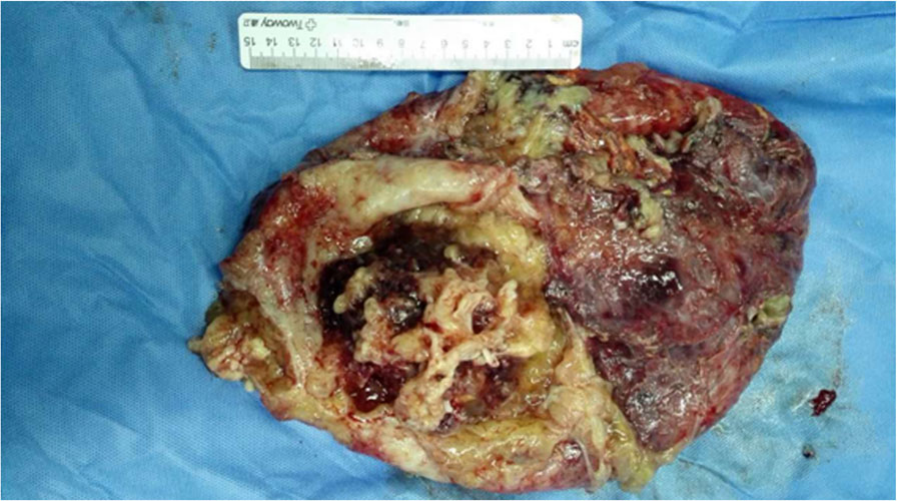
The mass was completely resected and it appeared to be a cystic-solid tumor

**Fig. 3:**
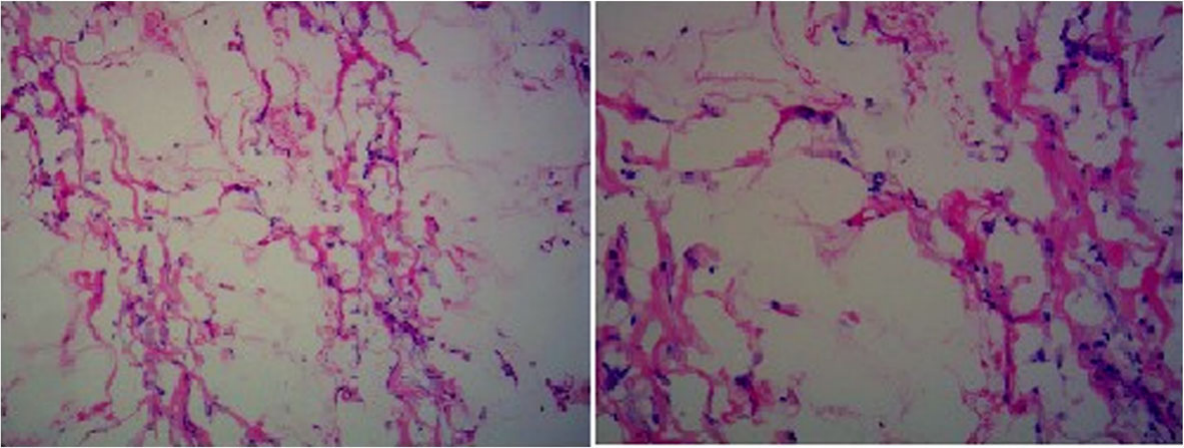
Intraoperative rapid pathology indicated low-grade mucinous neoplasm (×200)

**Fig. 4:**
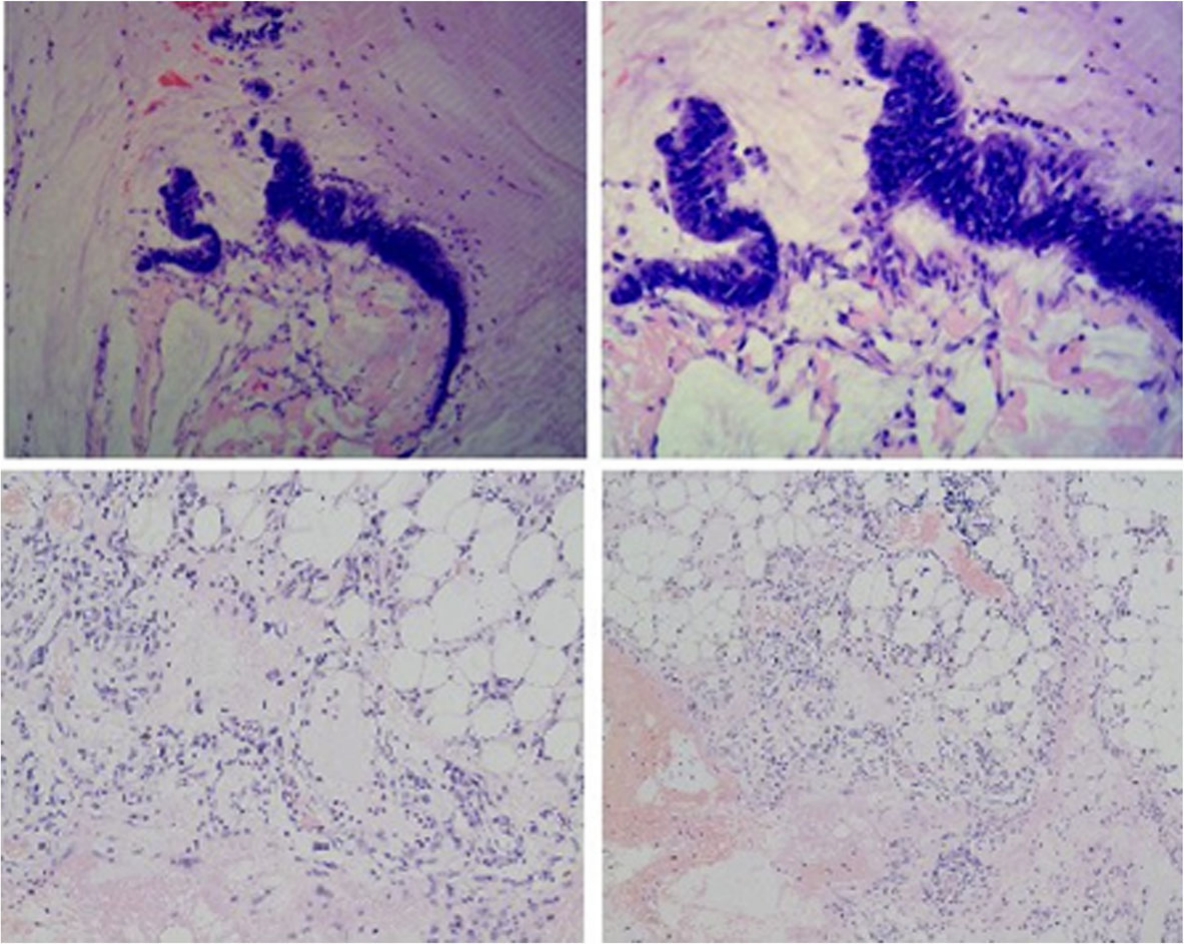
The final pathology report revealed a well-differentiated mucinous adenocarcinoma with mucin infiltrating into the soft tissue of the lump edge and omentum tissue (×200)

A 49-year-old Chinese Han man presented with abdominal distension of ten years’ duration. He stated that he did not experience abdominal pain, fever, chills, nausea, vomiting, constipation, or other discomfort symptoms. A systemic examination was within normal limits but an abdominal examination revealed a distended abdomen. Blood tests for tumor markers indicated increased ferritin (FERR; 2000 ug/ml; normal range, 15 to 200 ug/ml) and increased carbohydrate antigen 724 (CA724; 16.8 U/ml; normal range, 0 to 6 U/ml). A blood biochemistry test indicated decreased albumin (ALB; 24.8 g/L; normal range, over 40.5 g/L), decreased total protein (TP; 60.6 g/L; normal range, 65 to 85 g/L), decreased albumin to globulins ratio (A/G; 0.7; normal range, 1.2 to 2.4), decreased alanine aminotransferase (ALT; 7.9 U/L; normal range, 9 to 50 U/L), decreased aspartate aminotransferase (AST; 5.3 U/L; normal range, 15 to 40 U/L), decreased AST/ALT (0.7; normal range, 1 to 2), and decreased lactate dehydrogenase (LDH; 109 U/L; normal range, 135 to 226 U/L). Other routine laboratory tests were within normal range. He received an abdominal computed tomography (CT) scan which indicated a cystic-solid mass in his abdominal-pelvic cavity measuring 27.6 × 14.2 cm, and multi-node shadows in his omentum and partial mesenterium (Fig. 1). The diagnosis was an abdominal mass, the nature of which was still to be determined. He underwent exploratory laparotomy. During the surgery, a large mass was discovered which occupied the entire transverse colon zone. The boundary between the mass and surrounding tissue was unclear. After consultation, we decided to perform a lumpectomy. In order to avoid mass rupture, we adopted a blunt dissection method to handle it. Finally, the mass was resected completely without any leakage or rupture. After resection, the mass was roughly of round shape and measured 35 × 25 cm (Fig. 2). Approximately 6000 ml of fluid was aspirated from the mass, some of which appeared to be gelatinous material. Moreover, a fist-sized calcified tissue region was discovered in the mass. Intraoperative rapid pathology indicated a low-grade mucinous neoplasm, which are closely related to the appendix, and thus we considered the appendix as the origin for this mass (Fig. 3). Further exploration of his abdominal cavity did not reveal any additional lesions. Considering the size and the unclear boundary of the mass, the possibility of intra-abdominal metastasis was high. At that time, we decided that further treatment would depend on the final pathological outcome and thus a hemicolectomy was not performed. The final pathology report revealed a well-differentiated appendiceal mucinous adenocarcinoma with mucin infiltration into the soft tissue of the lump edge, and omentum tissue was infiltrated by mucinous nodules (Fig. 4). After several days of postoperative treatment, he recovered well and without any abdominal pain, bloating, or diarrhea. After recovery, he agreed to four courses of intraperitoneal infusion chemotherapy. At 15 months after surgery, he is in good condition and is being followed up.

## Discussion

MAA has an insidious onset; there are few or no symptoms early in the disease. With the development of the disease, patients may experience abdominal pain, bloating, abdominal mass, ascites, even intestinal obstruction, urinary tract obstruction, and other symptoms, over the duration of several months to several years [4]. MAA tumors are usually well differentiated and do not undergo metastatic spread until the late stages of the disease.

The treatment of choice is often surgical resection combined with adjuvant chemotherapy. The surgical approach for treating MAA depends on the specific circumstances of the tumor, such as tumor size, tumor location, histology of the tumor, and whether it is perforated or not, as well as the clinical presentation [5]. If the patient’s symptoms are not obvious, the lesions are confined to the mucosa (that is, carcinoma *in situ*). A simple appendectomy can also achieve the same effect as a right hemicolectomy. If the tumor cells have infiltrated through to the submucosa or the whole layer and become associated with regional lymph node metastasis, then a right hemicolectomy combined with omentectomy are often performed. If a histological examination shows the presence of mucinous peritoneal carcinomatosis, the patient should undergo cytoreductive surgery and intraperitoneal chemotherapy [6]. The most severe complication during such therapy is spontaneous or iatrogenic rupture, with development of a peritoneal pseudomyxoma (PMP) which significantly complicates the treatment and final outcome [7]. Once the preoperative diagnosis of the disease is MAA, surgeons need to avoid iatrogenic cyst rupture and peritoneal dissemination. Considering the serious consequence of iatrogenic rupture, we preserved the integrity of the cyst to avoid the potential proliferation of malignant mucin-producing cells in the abdominal cavity. In the present case, the final pathological result showed mucin infiltrating into the soft tissue of the lump edge and omentum tissue, which suggested that intra-abdominal metastasis had been formed. In this circumstance, radical surgery could not be carried out. Complete resection of the tumor plus postoperative formal chemotherapy was the best choice for our patient. For this case of a large appendiceal mucinous adenocarcinoma surgical resection plus adjuvant chemotherapy were effective treatments for a follow-up period of 15 months.

## Conclusions

Only very few cases of MAA have been reported and the present case is the largest MAA. Diagnosis of the disease is difficult due to its lack of specific symptoms and signs. Diagnosis is mainly achieved through the results of a CT scan. In our case, a frozen biopsy as a rapid pathological examination during surgery was helpful to guide further treatment. The appropriate surgical approach was chosen according to the histological type and differentiation degree of the specimen. Mucinous adenocarcinoma has a high risk of cavity planting; therefore, we should pay attention to protect the operative field and wound. Surgical resection plus adjuvant chemotherapy are currently an effective treatment with the aim of improving long-term survival.

## Abbreviations

A/G: Albumin to globulins ratio
ALB: Albumin
ALT: Alanine aminotransferase
AST: Aspartate aminotransferase
CA724: Carbohydrate antigen 724
CT: Computed tomography
FERR: Ferritin
LDH: Lactate dehydrogenase
MAA: Mucinous adenocarcinoma of the appendix
PMP: Peritoneal pseudomyxoma
TP: Total protein
